# Differences among the observers in the assessments of Japanese orthopedic association hip scores between surgeons and physical therapists and the correlations to patients’ reported outcomes after total hip arthroplasty

**DOI:** 10.1186/s12891-021-04980-5

**Published:** 2022-01-03

**Authors:** Hisaki Aiba, Nobuyuki Watanabe, Toshiaki Inagaki, Muneyoshi Fukuoka, Hideki Murakami

**Affiliations:** 1 Department of Orthopedic Surgery, Tosei General Hospital, 160, Nishioiwake-cho, Seto, Aichi 489-8642 Japan; 2 Department of Orthopedic Surgery, Nagoya City University, 1, Azakawasumi, Mizuho-cho, Mizuho-ku, Nagoya, Aichi 467-8601 Japan; 3 Department of Rehabilitation Medicine, Tosei General Hospital, 160, Nishioiwake-cho, Seto, Aichi 489-8642 Japan

**Keywords:** Total hip arthroplasty, JOA hips score, JHEQ, Physiotherapy, Patient-reported outcome, Clinician-reported outcome

## Abstract

**Background:**

We aimed to assess the utility of a clinician-reported outcome (the Japanese Orthopedic Association [JOA] hip score) as evaluated by clinicians and physiotherapists. This assessment was made by comparing these scores to those of the JOA hip disease evaluation questionnaire (JHEQ), which is a measurement of patient-reported outcomes after total hip arthroplasty.

**Methods:**

In this retrospective case-control study, 52 hips that underwent primary total hip arthroplasty were included in the analyses. The mean age of the participants was 66.8 years (sex, seven male and 45 female participants). The JOA hip score included four categories: pain, range of motion, ability to walk, and active daily living. The JHEQ included three categories: pain, movement, and mental health. These scores were evaluated preoperatively and postoperatively by clinicians or physiotherapists. Pearson’s correlation coefficients were utilized to analyze the association of the JOA hip scores to those of the JHEQ.

**Results:**

The JOA hip scores were determined by clinicians and physiotherapists (scores of 46.8 and 57.3, respectively) preoperatively and at 24 months (scores of 94.4 and 91.7, respectively) postoperatively. The JHEQ points were 28.8 and 66.2 preoperatively and at 24 months postoperatively, respectively. The correlation coefficients between the JOA hip and JHEQ scores were .66 and .69 preoperatively and .57 and .76 at 24 months postoperatively, as evaluated by clinicians and physiotherapists, respectively.

**Conclusions:**

Although the JHEQ scores were positively correlated to the JOA hip scores by clinicians and physiotherapists preoperatively and postoperatively, this study implies that clinicians may interpret the results in a way that might have been beneficial to them. To comprehend a patients’ health status, we should inclusively understand the varying range of information among different evaluators.

**Supplementary Information:**

The online version contains supplementary material available at 10.1186/s12891-021-04980-5.

## Background

With the aim to eliminate observational bias among clinicians for the evaluation of the quality of life (QOL) after surgeries, patient-reported outcomes measures (PROMs) should be regarded as indispensable measurements for the actual and subjective reflection of a patient’s condition and satisfaction. From 2002, the Swedish Hip Arthroplasty Registers, a nationwide arthroplasty registration conducted by the Swedish Orthopedic Society, initiated an observation program for total hip arthroplasty (THA) cases using PROMs [1, 2]. Moreover, the Food and Drug Administration and National Institute for Health and Care Excellence had recognized PROMs as essential methods for clinical investigators to measure the efficacy of medical intervention [3].

In contrast, clinician-reported outcomes (ClinROs) are commonly used as objective measures for surgical evaluations (i.e., Harris hip [4] and Merle d’Aubigné scores [5]), which are not long-listed questionnaires and reduce the patients’ subjective views of PROMs. However, it has not been fully established who among the various types of medical staff, including clinicians, nurses, or physical therapists, are suitable to extract ClinROs correctly. Moreover, although the significant differences between evaluations performed by physicians and patients have been noted [6], the range of discrepancy between PROMs and ClinROs still has not been evaluated.

In this study, based on the hypothesis that physiotherapists could evaluate the postoperative function from more independent viewpoints, we compared the results of ClinROs scored by surgeons or physiotherapists with those of the PROMs for patients with THA before and after surgery. Then, these assessments were compared and correlated to PROMs. In this study, the Japanese Orthopedic Association hip disease evaluation questionnaire (JHEQ, Supplement 1), which was specifically developed for the lifestyles of East Asian countries, was used as the PROM [7, 8], while the Japanese Orthopedic Association (JOA) hip score (Supplement 2) was used as the ClinRO [9].

## Methods

### Patients

From June 2012 to December 2017, we collected the data of patients who underwent THAs for degenerative hip disease cases and the regular assessments by PROMs and ClinROs at our hospital. The patients who had osteoarthritis, osteonecrosis, or rapidly destructive coxarthropathy and consented to participate into the study were included. We excluded those who underwent revision THA and acetabular reconstruction, as well as those who had rheumatic diseases and serious postoperative comorbidity complications.

All cases were reconstructed using cementless implants with the Revelation® hip system (DJO Global, Lewisville, TX, USA), SL-PLUS™ femoral hip system (Smith & Nephew, Hull, UK), MODULUS® femoral stem (Lima Corp., San Daniele, Italy), or C2® femoral stem (Lima Corp.). The reconstructions of the acetabular components were performed using the FMP® acetabular system (DJO Global) for the Revelation®, the R3Acetabular system® (Smith & Nephew) for the SL-PLUS®, and the Delta TT cup® (Lima Corp.) for the Modulus® and C2® femoral stems, respectively. All THAs were performed by one surgery team (organized by NW), by a modified Dall’s anterior-lateral approach [10].

### Patient-reported outcome measurement (PROM)

The JHEQ was evaluated as a PROM preoperatively and at 12 and 24 months postoperatively (Supplement 1). The JHEQ (maximum of 84 points) consisted of 20 questionnaires with subsections: pain, movement, and mental health (up to 28 points each) [7, 8]. At the same time, the visual analog scale (VAS) scores concerning the patients’ satisfaction regarding the surgical procedure were rated by them using an horizontal line of 100-mm long [11].

### Clinician-reported outcome (ClinRO)

Concerning ClinROs, the physicians and physical therapists who were engaged in the physical therapy and the rehabilitation programs after THA recorded the JOA hip score preoperatively and at 12 and 24 months postoperatively (Supplement 2). The JOA hip score had four categories for pain, range of motion (ROM), ability to walk, and activities of daily living (ADLs) (up to 40, 20, 20, and 20 points, respectively) [9].

### Statistical analysis

Shapiro–Wilk tests were performed for the confirmation of normal distributions of each characteristic. The ClinROs that were evaluated by the JOA hip scores before and after THA from different observers were compared using the Student’s *t*-test or the Mann–Whitney test in accordance with the results of the Shapiro–Wilk test. The correlations between the JOA hip and JHEQ scores were compared by Pearson’s correlation coefficients. The correlations between the VAS scores with the JOA hip or JHEQ scores were evaluated by Spearman’s correlation coefficients. A *P*-value <.01 was considered significant. The statistical package for the social sciences (SPSS ver. 24; IBM Corp., Armonk, NY, USA) was used for statistical analysis. The total sample size was determined on whether a correlation coefficient differed from zero (α = .01 [two-tailed], β = .20 and r = .45; target number = 52.7). Bland–Altman analysis and evaluation of the limit of agreement between the medical physicians and physical therapists were performed to assess the systemic bias [12, 13]. Identification of the fixed bias was evaluated based on whether the mean value of the difference differed significantly from 0 on the basis of a one-sample *t*-test. Moreover, the presence of proportional bias was investigated using the liner regression model.

### Role of the funding source

No funders participated in the design, conduct, or reporting of this study.

## Results

In this period, THAs were performed for degenerative hip disease cases at our institution (160 hips in total). We excluded revision THA cases (*n* = 17), acetabular reconstruction cases with acetabular support (*n* = 3), and cases of rheumatic diseases (*n* = 8). During the follow-up periods, we also omitted the cases of patients who had the following: required revision of THAs because of loosening (*n* = 1), postoperative deep located infection (*n* = 2), dislocation (*n* = 1), periprosthetic fracture (n = 1), and dementia, which would have affected the acquisition of accurate postoperative evaluation data (n = 1). Thirty patients dropped out from the routine surveys after THA in our hospital. Among 96 cases, a total 52 of patients (cases of osteoarthritis [*n* = 46], osteonecrosis [n = 4], and rapidly destructive coxarthropathy [*n* = 2]) agreed to participate in this study and completed the consecutive questionnaires. The average age was 66.8 (standard deviation [SD], 8.9) years. In total, THAs were performed for 45 and seven joints of women and men, respectively. The median operation time was 98 min, the median total amount of surgical bleeding was 250 mL, and the average body mass index was 23.5 kg/m^2^. The details of the patients’ characteristics or implants’ information are presented in Tables 1 and 2.

**Table 1:** Patients’ characteristics

Female vs. male	45 vs. 7
Right vs. left hip surgery	30 vs. 22
Age (years; mean, standard deviation)	66.8 ± 8.9
Body mass index (kg/m^2^; mean, standard deviation)	23.5 ± 3.4
Operation time (minutes; median, range)	97.5 (76–168)
Total amount of bleeding (mL; median, range)	250.0 (10–660)
Harris Hip scores, preoperatively (points [total 100]; mean, standard deviation)	54.0 ± 12.0

**Table 2:** Implants’ information

Femoral stem	Acetabular components	Number
Revelation® hip system	FMP® acetabular system	10
SL-PLUS™ femoral hip system	R3 Acetabular system®	36
MODULUS® femoral stem	Delta TT cup®	5
C2® femoral stem	Delta TT cup®	1

### ClinROs between different observers

Preoperatively, the median JOA hip scores, as assessed by physicians, were 46.5 points in total: 10, 10, 10, and 10 points for pain, ROM, ability to walk, and ADL, respectively. In contrast, the median JOA hip scores, as assessed by physical therapists, were 57.0 points in total; 20, 14, 10, and 12 points for pain, ROM, ability to walk, and ADL. Therefore, the JOA hip scores evaluated by the orthopedic surgeons were significantly lower (*P* < .01) than those evaluated by the physical therapists, except for the scores for ability to walk (Table 3).

**Table 3:** Clinician-reported outcome from different observers

		Physicians (median, IQR)	Therapists (median, IQR)	*P*-value
JOA hip scores (preoperative)	Pain (/40 points)	10 (10–20)	20 (10–30)	.003
	ROM (/20 points)	10 (8–12.8)	14 (12–16	<.001^*1^
	Walk (/20 points)	10 (5–15)	10 (5–15)	.912
	ADL (/20 points)	10 (8–12)	12 (10–14)	<.001
	Total (/100 points)	46.5 (37.3–57)	57.0 (41.8–69.0)	<.001^*1^
JOA hip scores (12 months, postoperatively)	Pain (/40 points)	39 (38–40)	37 (35–40)	.003
	ROM (/20 points)	20 (18–20)	18 (17–20)	<.001
	Walk (/20 points)	20 (15–20)	18 (15–20)	.004
	ADL (/20 points)	16 (16–19.5)	18 (14–20)	.614
	Total (/100 points)	94.0 (90.3–98.0)	92 (81.8–98.0)	<.001^*1^
JOA hip scores (24 months, postoperatively)	Pain (/40 points)	39 (40–40)	38 (35–40)	.004
	ROM (/20 points)	20 (20–20)	19 (17–20)	<.001
	Walk (/20 points)	20 (15–20)	20 (15.6–20)	.709
	ADL (/20 points)	18 (16–20)	18 (16–20)	.504
	Total (/100 points)	96.0 (91.0–98.0)	94.5 (88.0–98.0)	.004^*1^

*ADL* activities of daily living, *JOA* Japanese Orthopedics Association, *IQR* interquartile range, *ROM* range of motion. All values are presented as medians and IQRs. The differences were evaluated by the Mann–Whitney test. In cases of normal distribution, the differences were evaluated by Student’s *t*-test. *1, normal distribution

After THA, the mean total JOA hip score improved and gradually restored over time from preoperative scores to 94.0 and 92.0 (12 months, *P* < .001), and to 96.0 and 94.5 (24 months, *P* = .004) postoperatively, as evaluated by surgeons and physical therapists, respectively. Unlike the preoperative evaluations, several subcategories of JOA hip scores, including pain, ROM, ability to walk (only 12 months), and total scores, were significantly overestimated by the orthopedic surgeons (Table 3).

Preoperatively, the Bland–Altman analysis suggested the downward fixed bias in the total JOA hip scores evaluated by physicians about 10 points (*P* < .001). On the contrary, the Bland–Altman analysis suggested the presence of upward fixed bias in the score evaluated by physicians (12 months, 4.3 points (P < .001); 24 months, 2.8 points (*P* = .006)). Moreover, there were proportional errors (R = -.46, P < .001; R = -.48, P < .001; 12 and 24 months, respectively; Supplement 3).

### Correlations of ClinROs and PROMs

Preoperatively, the median total JHEQ score was 30.0 points (pain, 9; movement, 5; mental health, 12). At 12 months postoperatively, the mean total JHEQ score was 67.5 points (pain, 28; movement, 18; mental health, 24.5) (Supplement 4). Then, at 24 months postoperatively, the total JHEQ score was 67.5 points (pain, 27; movement, 18.5; mental health, 25). The correlations of the total JOA and JHEQ scores at preoperative periods were .66 and .69 (evaluated by physicians and therapists, respectively; Table 4 and Supplement 5). Moreover, at 24 months postoperatively, the correlations of the total JOA and JHEQ scores were .57 and .76 (evaluated by physicians and therapists, respectively; Table 4 and Supplement 5).

**Table 4:** Correlation between the JOA hip and JHEQ scores

Pre-operation	Median, IQR	Observers	Correlation				
			JOA-pain	JOA-ROM	JOA-walk	JOA-ADL	JOA-total
JHEQ-pain	9.0 (6.0–13.8)	Physicians	.46*	.30	.23	.42*	.53**
		Therapists	.60**	.27	.31	.40*	.58**
JHEQ-movement	5 (2.0–9.0)	Physicians	.29	.43*	.54**	.57**	.62**
		Therapists	.50**	.45*	.57**	.68**	.68**
JHEQ-mental	12 (7.0–18.8)	Physicians	.26	.44*	.52**	.47**	.57**
		Therapists	.46*	.29	.49**	.50**	.57**
JHEQ-total	30.0 (14.0–40.0)	Physicians	.39	.45*	.49**	.55**	.66**
		Therapists	.60**	.37	.52**	.59**	.69**
Post-operation (24 months)	Median, IQR	Observers	Correlation				
			JOA-pain	JOA-ROM	JOA-walk	JOA-ADL	JOA-total
JHEQ-pain	27.0 (22.0–28.0)	Physicians	.32	.11	.32	.42*	.45*
		Therapists	.39*	.21	.63**	.60**	.66**
JHEQ-movement	18.5 (12.0–24.0)	Physicians	.37	.19	.37	.48**	.54**
		Therapists	.43*	.45*	.51**	.67**	.71**
JHEQ-mental	25.0 (20.3–27.8)	Physicians	.31	.06	.44**	.59**	.54**
		Therapists	.36	.23	.66**	.55**	.65**
JHEQ-total	67.5 (57.0–78.3)	Physicians	.38*	.10	.42*	.56**	.57**
		Therapists	.44**	.35	.66**	.69**	.76**

*ADL* activities of daily living, *IQR* interquartile range, *JHEQ* Japanese orthopedic association hip disease evaluation questionnaire, *JOA* Japanese Orthopedics Association, *ROM* range of motion, *P < .01, ** *P* < .001. The scatter plots of the distribution of JOA hip scores and JHEQ preoperatively and 24 months after THA in the Supplement 5

### Correlations of patients’ satisfaction and pain measured by VAS with JOA hip scores or JHEQs

As representative continuous values of the outcomes, the VAS-satisfaction for hip joints were evaluated. Preoperatively, the median VAS-satisfaction was 13 points. After THA, these complaints were resolved to more than a median of 95 points within 12 months. When comparing the relationships between VAS-satisfaction and JOA or JHEQ, the correlations were calculated by Spearman’s correlations. Therefore, VAS-satisfaction was found to be highly correlated to the total JHEQ score (Table 5, Supplement 6) after comparing to the JOA hip score preoperatively and moderate correlated at 12 and 24 months after THA.

**Table 5:** Correlation between JOA hip scores and VAS

		Score (median, IQR)	JOA-physicians	JOA-therapists	JHEQ-total
VAS-satisfaction (/100)	Pre-operation	13 (2–33)	.32	.44*	.61**
	Post-operation (12 months)	95 (83–100)	.44*	.45*	.55**
	Post-operation (24 months)	96.5 (86–100)	.44*	.43*	.71**

*ADL* activities of daily living, *JHEQ* Japanese orthopedic association hip disease evaluation questionnaire, *JOA* Japanese Orthopedics Association, *ROM* range of motion, *VAS* visual analog scales, * *P* < .01, ** *P* < .001. The scatter plots of the distribution of JOA hip scores /JHEQ and VAS-satisfaction preoperatively and 24 months after THA in the Supplement 6

## Discussion

In this study, we first described postoperative evaluation after THA with the ClinROs, as evaluated by different observers, and analyzed the relationships between the PROMs and ClinROs (customized measurements for the East Asian populations), and the JOA hip and JHEQ scores. Interestingly, we found that physical therapists could substitute the essential evaluators for the ClinROs from more independent viewpoints compared with physicians.

For the assessment of postoperative function, pain, satisfaction, or QOL, more reliable patient-oriented evaluation criteria are desired. As a representation of changes for multiple symptoms, these criteria clarify the impact of treatment and enhance the interpretation of clinical studies for clinicians [14]. To date, the Short-Form 36-Item Health Survey (SF-36) [15], Western Ontario and McMaster Universities Osteoarthritis Index (WOMAC) [16], and Oxford Hip Score [17] are generally used as PROMs [7, 8]. The JHEQs were designed to adjust for the sedentary Asian lifestyle, which requires deep hip flexion for several activities, including sitting upright and usage of traditional toilets [18]. Moreover, the JHEQ covered the subjective dissimilarities of the patients, which are difficult to determine from the objective examinations. Thus, the JHEQ provides meaningful information in the actual clinical setting.

Originally, Seki et al. reported that the JHEQ in cases of osteoarthritis or necrosis presented excellent reliability (intraclass correlation coefficient [ICC] > .8), while the JOA hip score was reliable in Japanese patients with osteoarthritis (Cronbach’sα test = .70) [8]. Moreover, in a previous work, we reported that the ICC (1.2) in the JHEQ subgroup of patients with labral tear was .88 in all categories (pain, .85; movement, .89; mental, .8), while the Cronbach’s α test result was .94 in all categories (pain, .92; movement, .94; mental, .89 in subcategory) [19]. Based on the preceding sufficient results, we did not duplicate the reliability tests in our patients.

For various types of illness, PROMs and ClinROs had been compared and disagreements among numerous studies have been reported. Generally, patient-reported symptoms provided an independent patients’ perspective on the treatment benefit and the expected risk, which occasionally exceeds the clinicians’ expectations. Flores et al. [20] reported that patients with rectal cancer who were treated with chemo-radiotherapy described the presence of diarrhea and proctitis more often than when recorded by clinicians throughout treatment. For patients with breast cancer treated with radiotherapy, Mukesh et al. [21] reported that moderate-to-severe toxicity was underestimated as low toxicity by clinicians, and the overall concordance between clinicians and patients was not sufficient. In this study, as reliable values, VAS-satisfaction was more precisely correlated to the JHEQ score compared with the JOA hip scores.

To our knowledge, this is the first study to compare the JOA hip scores recorded by surgeons with those recorded by physiotherapists. There were significant differences between the JOA hip scores recorded by physicians and physiotherapists for approximately all investigations preoperatively and postoperatively. Nevertheless, the maximum differences might not be a critical discrepancy in clinical settings (only < 5 points), but this study indicated that the JOA scores were overestimated after THA by clinicians. However, the reasons for the systemic tendencies were not fully elucidated; clinicians reported upward postoperative scores without consciousness (a sort of rater bias). In contrast, preoperative JOA-pain, ROM, and ADL scores were underestimated by clinicians. These inclinations might have been a bias at the time of selection of patients for THA; securing a stable number of cases for surgery is important for the clinicians. These data suggested the physical therapists can correctly report the pre- and postoperative functions from more objective viewpoints.

Moreover, the correlation coefficients between the JOA hip and JHEQ scores were higher for physiotherapists than for clinicians, especially for the preoperative JOA-pain/JHEQ-pain, preoperative JOA-ADL/JHEQ-movement, postoperative JOA-walk/JHEQ-movement, and postoperative JOA-ADL/JHEQ-movement. These findings were partially related to the fact that well-educated rehabilitation staff can more accurately evaluate the patients’ status than physicians with closer relationships to the patients and provide more open-minded circumstances to present their problems before and after operation. The assessment by physiotherapists might support clinicians with a more objective perception to exclude observational bias of patients’ status. As objective observers, ClinROs by physiotherapists should be considered in evaluating postoperative outcomes.

This study had several limitations. First, the number of patients in this study was small and the institutional difference should be noted. These differences included the conditions of the surgeries and the experience of the young physicians in residency and medical staff who evaluated JOA hip scores. Second, we excluded technically difficult cases (revision THA, acetabular reconstruction cases with acetabular support, and extensive infection cases), for which, postoperative functions are not generally guaranteed and dispersed. Third, the presence of bias should be noted; especially, patients who were willing to participate in the study and answer the questionnaires were mainly selected for this study. Moreover, relatively low response rates to this study might have influenced the results. This was attributed to the accessibility to our institution and introduction to clinics located near the participants’ dwelling places. However, the response rate was not intentional and could not have affected the results of this study.

## Conclusion

The JHEQ score was correlated to the JOA hip score, as measured by clinicians and physiotherapists. However, this study implicated that rater bias might have influenced the results. To determine a patient’s status, it is recommended that the various selections of information collected among different observers should be inclusively understood and evaluated.

## Supplementary Information

**Additional file 1.** Japanese Orthopedic Association Hip-Disease Evaluation Questionnaire: A Patient-Based Evaluation Tool for Hip-Joint Disease” [22].

**Additional file 2.** Japanese Orthopedic Association hip score [23].

**Additional file 3.** Bland–Altman in the total JOA hip scores.

**Additional file 4.** Correlation between JOA hip scores and JHEQ scores in 12 months.

**Additional file 5.** The scatter plots of the distribution of JOA hip scores and JHEQ preoperatively and 24 months after THA.

**Additional file 6.** The scatter plots of the distribution of JOA hip scores /JHEQ and VAS-satisfaction preoperatively and 24 months after THA.

## Data Availability

The datasets used and/or analyzed during the current study are available from the corresponding author on reasonable request.

## Abbreviations

ADL: Active daily living
ClinRO: Clinician-reported outcome
FDA: Food and Drug Administration
JHEQ: Japanese Orthopedic Association hip disease evaluation questionnaire
JOA: Japanese Orthopedic Association
OHS: Oxford Hip Score
PROM: Patient-reported outcomes measure
QOL: Quality of life
ROM: Range of movement
THA: Total hip arthroplasty
NICE: National Institute for Health and Care Excellence
SF-36: Short-Form 36-Item
SD: Standard deviation
WOMAC: Western Ontario and McMaster Universities Osteoarthritis Index
