# Supplementary material for: Differences among the observers in the assessments of Japanese orthopedic association hip scores between surgeons and physical therapists and the correlations to patients’ reported outcomes after total hip arthroplasty
Source: BMC Musculoskelet Disord. 2022 Jan 3;23:27. doi: 10.1186/s12891-021-04980-5 (PMC8725241; doi:10.1186/s12891-021-04980-5)
Supplement: Supplementary file 1 — Additional file 1. Japanese Orthopedic Association Hip-Disease Evaluation Questionnaire: A Patient-Based Evaluation Tool for Hip-Joint Disease” [22]. [file 12891_2021_4980_MOESM1_ESM.pdf]

Japanese Orthopedic Association Hip Disease Evaluation Questionnaire

The following questionnaire asks you to describe your hip-joint condition, including difficulties you may encounter in daily life. Your honest appraisals will help us improve treatment for patients in the future. Thank you for taking the time to fill out this questionnaire.

Guidelines

① Please make sure that your answers to the questions pertain to your hip-joint condition within the past 3 months.

② Please refer to the examples below in answering the questions.

③ Please try answer all questions. However, if you find some questions difficult to answer, you may skip them.

④ In this questionnaire, there are two types of questions:

I. The first type asks you to place an X on a line to indicate your answer.

II. The second type asks you place a check mark in the box that applies to you.

Type I Question Example.

＜Important Point＞

Place an X on the line that corresponds to your answer.

Good Examples

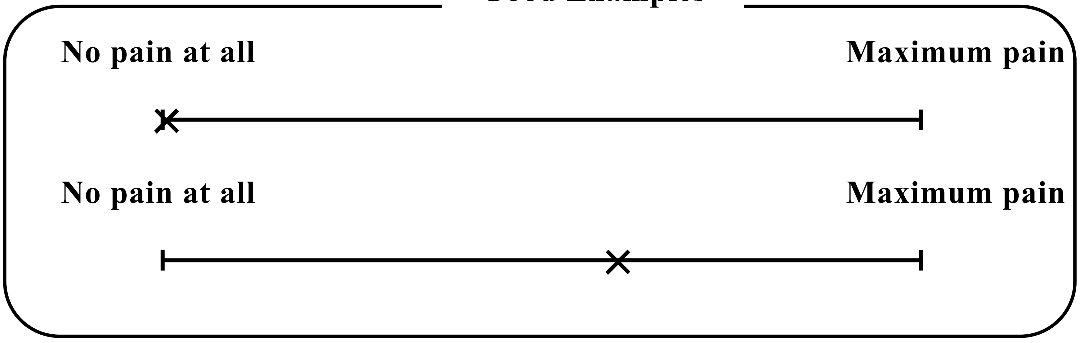

In this case the X is not on the line, but beside or above it.

Bad Examples

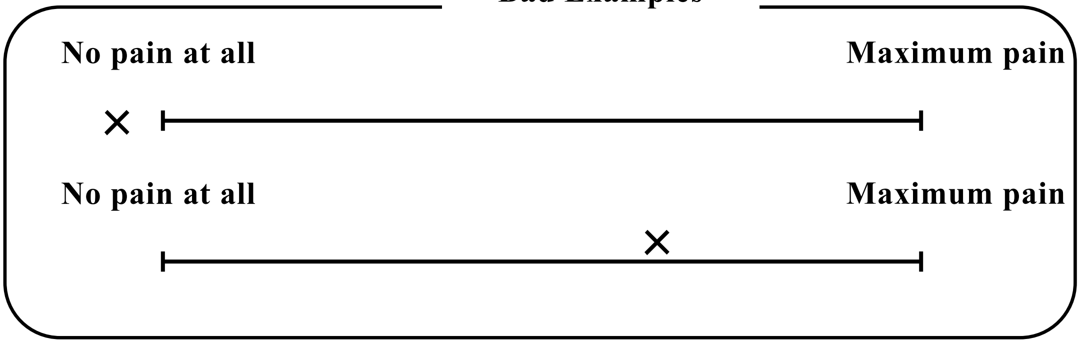

Type II Question Example

＜Important Point＞

Please check ONE box that most applies to you.

> **Good example:**
> **1 box is checked.**
>
> **Bad example:**
> **2 boxes are checked.**

		Strongly Agree	Agree	Uncertain	Disagree	Strongly Disagree
**Good example**	Even when I am at rest, my hip is painful.	☐	☑	☐	☐	☐
**Bad example**	Even when I am at rest, my hip is painful.	☑	☑	☐	☐	☐

The Questionnaire Begins Here

*Please respond regarding your current (within the last 3 months) hip condition.

1. In general, rate your current level of satisfaction as it pertains to your hip condition.

Place an X on the continuum, where the left side represents complete satisfaction and the right represents complete dissatisfaction.

Complete Satisfaction **Complete Dissatisfaction**

2. How severe is your hip pain?

Place an X on the continuum of pain, where the left side represents no pain at all and the right side represents maximum pain.

＜Right Side Hip＞

No Pain at all **Maximum Pain**

＜Left Side Hip＞

No Pain at all **Maximum Pain**

Please place a check mark in the box for each question that applies to you.

		Strongly Agree	Agree	Uncertain	Disagree	Strongly Disagree
**1.** Even when I am at rest, my hip is painful.	**Right**	☐	☐	☐	☐	☐
	**Left**	☐	☐	☐	☐	☐
**2.** My hip is painful when I sit in a chair.	**Right**	☐	☐	☐	☐	☐
	**Left**	☐	☐	☐	☐	☐
**3.** I feel pain in my hip when I start to move.	**Right**	☐	☐	☐	☐	☐
	**Left**	☐	☐	☐	☐	☐
**4.** I cannot move my hip joint freely because of the pain.	**Right**	☐	☐	☐	☐	☐
	**Left**	☐	☐	☐	☐	☐
**5.** The pain in my hip joint prevents me from moving with strength.	**Right**	☐	☐	☐	☐	☐
	**Left**	☐	☐	☐	☐	☐
**6.** Because of hip-joint pain, I occasionally cannot sleep well.	**Right**	☐	☐	☐	☐	☐
	**Left**	☐	☐	☐	☐	☐
**7.** It is difficult for me to climb up and down stairs.		☐	☐	☐	☐	☐
**8.** It is difficult for me to get up from the floor and tatami.		☐	☐	☐	☐	☐
**9.** It is difficult for me to squat.		☐	☐	☐	☐	☐
**10.** It is difficult for me to use a Japanese-style toilet.		☐	☐	☐	☐	☐

		Strongly Agree	Agree	Uncertain	Disagree	Strongly Disagree
**11.** It is difficult to get in and out of a bathtub.		☐	☐	☐	☐	☐
**12.** It is difficult to cut my toenails.	**Right**	☐	☐	☐	☐	☐
	**Left**	☐	☐	☐	☐	☐
**13.** It is difficult to put on my socks.	**Right**	☐	☐	☐	☐	☐
	**Left**	☐	☐	☐	☐	☐
**14.** Because of hip-joint disease, I sometimes get irritated or feel nervous.		☐	☐	☐	☐	☐
**15.** Because of hip-joint disease, I feel dispirited and avoid going out.		☐	☐	☐	☐	☐
**16.** Because of hip-joint disease, I feel anxiety about my livelihood/daily life.		☐	☐	☐	☐	☐
**17.** Because of hip-joint disease, I feel dissatisfied with my health.		☐	☐	☐	☐	☐
**18.** My hip-joint condition deeply affects my well-being.		☐	☐	☐	☐	☐
**19.** Because of hip joint disease, it is difficult for me to actively undertake various things.		☐	☐	☐	☐	☐
**20.** Because of hip-joint pain, sometimes participation in local events and neighborhood relationships does not go smoothly for me.		☐	☐	☐	☐	☐

Thank you for completing this questionnaire.

The change of the JHEQ is prohibited. © 2011 The Japanese Orthopaedic Association
