# Supplementary material for: Differences among the observers in the assessments of Japanese orthopedic association hip scores between surgeons and physical therapists and the correlations to patients’ reported outcomes after total hip arthroplasty
Source: BMC Musculoskelet Disord. 2022 Jan 3;23:27. doi: 10.1186/s12891-021-04980-5 (PMC8725241; doi:10.1186/s12891-021-04980-5)
Supplement: Supplementary file 2 — Additional file 2. Japanese Orthopedic Association hip score [23]. [file 12891_2021_4980_MOESM2_ESM.docx]

**Appendix 2. Japanese Orthopedic Association hip score**

| **A. Pain** | (Full mark = 40 points) | |
| --- | --- | --- |
|  | Right | Left |
| None^a^ | 40 | 40 |
| Ignores^b^ | 35 | 35 |
| Slight^c^ | 30 | 30 |
| Moderate^d^ | 20 | 20 |
| Severe^e^ | 10 | 10 |
| Unbearable^f^ | 0 | 0 |

^a^ No pain and/or no complaints relating to hip joint

^b^ No pain. Inconstant symptoms including weary feeling or dullness

^c^ No spontaneous pain. Some pain when walking (including slight pain when starting to walk or after walking for long distance)

^d^ No spontaneous pain. Some pain when walking but which disappears quickly after a short rest

^e^ Spontaneous pain. Pain is severe when attempting to walk; it decreases after a rest

^f^ Continuous pain during rest and/or at night

| **B. Range of motion** | (Full mark = 20 points) | |
| --- | --- | --- |
|  | Right | Left |
| Flexion arc | 0–12 | 0–12 |
| Abduction arc | 0–8 | 0–8 |

Scores are detemined by multiplying 10° of motion in each arc by 1 point in flexion and 2 points in abduction. Range of contracture should be subtracted

A flexion are more than 120° is detemined as 12 points and the abduction are more than 30° as 8 points

Either flexion and abduction is measured in neutral position on rotation and described by its arc by passive motion

| **C. Ability to walk** | (Full mark = 20 points) |
| --- | --- |
| Normal^a^ | 20 |
| Slight limp^b^ | 18 |
| Mild limp^c^ | 15 |
| Severe limp^d^ | 10 |
| Difficult to walk^e^ | 5 |
| Impossible^f^ | 0 |

^a^ Able to walk long distance without limp. Able to walk fast

^b^ Able to walk long distance including walking with a slight limp. Able to walk fast

^c^ Able to walk 30 min or 2 km without support. Mild limp

^d^ Able to walk 10–15 min or 50 m without support

^e^ Able to do household activities. Difficult to do outdoor activities. Difficult to walk outdoors without bilateral supports

^f^ Impossible or almost impossible to walk

| **D. Activities of daily life** | (Full mark = 20 points) | | |
| --- | --- | --- | --- |
|  | Normal | Difficult | Impossible |
| Sitting on chair | 4 | 2 | 0 |
| Standing work (including housework)^a^ | 4 | 2 | 0 |
| Squatting, standing up from sitting on the floor^b^ | 4 | 2 | 0 |
| Going up and down stairs^c^ | 4 | 2 | 0 |
| Getting into car or entering public transport | 4 | 2 | 0 |

^a^ Able to continue longer than 30 min. Needs to take a rest — score as “difficult.” Unable to continue longer than 5 min — score as “impossible”

^b^ Support needed — score as “difficult”

^c^ Handrail needed — score as “difficult”
