# Supplementary material for: Differences among the observers in the assessments of Japanese orthopedic association hip scores between surgeons and physical therapists and the correlations to patients’ reported outcomes after total hip arthroplasty
Source: BMC Musculoskelet Disord. 2022 Jan 3;23:27. doi: 10.1186/s12891-021-04980-5 (PMC8725241; doi:10.1186/s12891-021-04980-5)
Supplement: Supplementary file 3 — Additional file 3. Bland–Altman in the total JOA hip scores. [file 12891_2021_4980_MOESM3_ESM.docx]

**Appendix 3. Bland–Altman in the total JOA hip scores**

The difference was calculated using the points of the JOA hips score as evaluated by the physicians minus the score after evaluation by the physiotherapists. The mean values indicate the average of the JOA hip score as evaluated by physicians and physiotherapists. The black line indicates the mean and the dot lines indicate the 95% confidence intervals.
