# Supplementary material for: Differences among the observers in the assessments of Japanese orthopedic association hip scores between surgeons and physical therapists and the correlations to patients’ reported outcomes after total hip arthroplasty
Source: BMC Musculoskelet Disord. 2022 Jan 3;23:27. doi: 10.1186/s12891-021-04980-5 (PMC8725241; doi:10.1186/s12891-021-04980-5)
Supplement: Supplementary file 4 — Additional file 4. Correlation between JOA hip scores and JHEQ scores in 12 months. [file 12891_2021_4980_MOESM4_ESM.docx]

**Appendix 4. Correlation between the JOA hip and JHEQ scores at 12 months postoperatively**

| Post-operation  (12 months) | Median,  IQR | Observers | Correlation | | | | |
| --- | --- | --- | --- | --- | --- | --- | --- |
|  |  |  | JOA-pain | JOA-ROM | JOA-walk | JOA-ADL | JOA-total |
| JHEQ-pain | 28.0  (22.3–28.0) | Physicians  Therapists | .58**  .68** | .17  .23 | .50**  .60** | .51**  .62** | .62**  .73** |
| JHEQ-movement | 18.0  (12.3–21.0) | Physicians  Therapists | .40*  .52** | .30  .52** | .48**  .61** | .61**  .73** | .64**  .75** |
| JHEQ-mental | 24.5  (18.3–28.0) | Physicians  Therapists | .33  .56** | .11  .29 | .60**  .68** | .66**  .65** | .65**  .73** |
| JHEQ-total | 67.5  (56.0–75.5) | Physicians  Therapists | .47**  .64** | .21  .39* | .58**  .70** | .66**  .74** | .70**  .81** |

ADL, activities of daily living; JHEQ, Japanese orthopedic association hip disease evaluation questionnaire; JOA, Japanese Orthopedics Association; ROM, range of motion, *P<.01, ** P<.001.
