# Supplementary material for: Differences among the observers in the assessments of Japanese orthopedic association hip scores between surgeons and physical therapists and the correlations to patients’ reported outcomes after total hip arthroplasty
Source: BMC Musculoskelet Disord. 2022 Jan 3;23:27. doi: 10.1186/s12891-021-04980-5 (PMC8725241; doi:10.1186/s12891-021-04980-5)
Supplement: Supplementary file 5 — Additional file 5. The scatter plots of the distribution of JOA hip scores and JHEQ preoperatively and 24 months after THA. [file 12891_2021_4980_MOESM5_ESM.docx]

**Appendix 5. The scatter plots of the distribution of JOA hip scores and JHEQ preoperatively and 24 months after THA.**

The relationships between total JOA hip scores and JHEQ preoperatively and 24-months postoperatively were shown. The blue lines and dots indicated scores assessed by physicians and estimated regression lines. Likewise, the red lines and dots indicated scores assessed by therapists and estimated regression lines.

JHEQ, Japanese orthopedic association hip disease evaluation questionnaire; JOA, Japanese Orthopedics Association.
